# Relationship of socio-demographics, comorbidities, symptoms and healthcare access with early COVID-19 presentation and disease severity

**DOI:** 10.1186/s12879-021-05764-x

**Published:** 2021-01-09

**Authors:** Laura Vaughan, Darlene Veruttipong, Jonathan G. Shaw, Noemie Levy, Lauren Edwards, Marcy Winget

**Affiliations:** 1 Division of Primary Care & Population Health, Stanford University School of Medicine, 1265 Welch Rd., Mail Code 5475, Stanford, CA 94305 USA; 2 Stanford Primary Care Los Altos, 960 N San Antonio Rd #101, Los Altos, CA 94022 USA; 3 Evaluation Sciences Unit, Department of Medicine, Stanford University School of Medicine, Stanford, USA; 4 Stanford University School of Medicine, 291 Campus Drive, Stanford, CA 94305 USA

**Keywords:** COVID-19, Race, Socio-demographics, Comorbidities, Symptoms

## Abstract

**Background:**

COVID-19 studies are primarily from the inpatient setting, skewing towards severe disease. Race and comorbidities predict hospitalization, however, ambulatory presentation of milder COVID-19 disease and characteristics associated with progression to severe disease is not well-understood.

**Methods:**

We conducted a retrospective chart review including all COVID-19 positive cases from Stanford Health Care (SHC) in March 2020 to assess demographics, comorbidities and symptoms in relationship to: 1) their access point of testing (outpatient, inpatient, and emergency room (ER)) and 2) development of severe disease.

**Results:**

Two hundred fifty-seven patients tested positive: 127 (49%), 96 (37%), and 34 (13%) at outpatient, ER and inpatient, respectively. Overall, 61% were age < 55; age > 75 was rarer in outpatient setting (11%) than ER (14%) or inpatient (24%). Most patients presented with cough (86%), fever/chills (76%), or fatigue (63%). 65% of inpatients reported shortness of breath compared to 30–32% of outpatients and ER patients. Ethnic/minority patients had a significantly higher risk of developing severe disease (Asian OR = 4.8 [1.6–14.2], Hispanic OR = 3.6 [1.1–11.9]). Medicare-insured patients were marginally more likely (OR = 4.0 [0.9–17.8]). Other factors associated with developing severe disease included kidney disease (OR = 6.1 [1.0–38.1]), cardiovascular disease (OR = 4.7 [1.0–22.1], shortness of breath (OR = 5.4 [2.3–12.6]) and GI symptoms (OR = 3.3 [1.4–7.7]; hypertension without concomitant CVD or kidney disease was marginally significant (OR = 2.3 [0.8–6.5]).

**Conclusions:**

Early widespread symptomatic testing for COVID-19 in Silicon Valley included many less severely ill patients. Thorough manual review of symptomatology reconfirms the heterogeneity of COVID-19 symptoms, and challenges in using clinical characteristics to predict decline. We re-demonstrate that socio-demographics are consistently associated with severity.

## Background

During the week of February 23, 2020, community spread of the virus that causes COVID-19 was reported in California. On March 7 the Centers for Disease Control (CDC) reported 213 confirmed cases in the US [1]. Early data on COVID-19 symptoms is primarily from the inpatient setting and therefore skews towards the more severely ill, leaving many knowledge gaps around characteristics of the disease in the ambulatory population.

Health professionals have struggled to identify who is most at risk of severe disease, as COVID-19’s variable and wide-ranging symptoms make symptom-based diagnosis and prediction of progression to severe disease a challenge [2]. Instead, as community spread of COVID-19 has exponentially increased and testing has scaled up, race and ethnicity have consistently been found to predict hospitalization and mortality [1, 3, 4]. The complexities underlying these health disparities are uncertain, and undoubtedly include a mix of social, economic, access, and behavioral factors [3]. Moreover, certain comorbidities (including diabetes, heart disease, chronic kidney disease, and obesity) are now known to strongly predict COVID-19 hospitalization [5]; most of these comorbidities have disparate prevalence by race/ethnicity, but are not alone sufficient to explain racial/ethnic disparities in COVID-19’s impacts.

Amidst this complex interplay of factors, the ambulatory presentation of milder COVID-19 disease, and which characteristics might predict progression to severe disease, remains poorly understood. Widespread testing and clinical documentation of ambulatory cases is necessary to fill in our understanding of the spectrum of COVID-19 disease. In March 2020, Stanford University in Santa Clara County was the first California healthcare system to institute drive-through testing available to anyone in the outpatient setting with COVID-19 symptoms, regardless of their insurance status [6].

Stanford testing guidelines in March 2020 were relatively broad and symptom based, allowing testing of anyone with new-onset fever, cough, sore throat, or shortness of breath or flu-like symptoms in the preceding 14 days. By comparison, the CDC guidelines at the time limited testing to those with lower respiratory infection, travel to known high risk regions, and known or suspected close contact with COVID-19+ individuals. As a result of its criteria, in its first month of testing, Stanford included a broad population of less severely ill patients than most prior reported cohorts. Moreover, the tested cohort presents unique socio-demographic diversity, as Silicon Valley has both wide disparities in income and unique racial/ethnic diversity—Santa Clara and San Mateo counties’ have high population representation of Asian (39 and 30% respectively) and Hispanic/Latinx (36 and 24%), but relative under-representation of African American/Black (< 3%) [7, 8].

We conducted a detailed chart review of COVID-19 positive patients presenting in the first month of wide-scale symptomatic testing to explore the patterns of sociodemographic, co-morbid conditions, and symptomatology to further our understanding of the disease.

## Methods

### Setting

Stanford Health Care (SHC) is an academic health system in Silicon Valley. It began testing for COVID-19 on March 4, 2020 using a reverse-transcriptase polymerase chain reaction (RT-PCR) diagnostic test developed by Stanford’s clinical virology laboratory. The diagnostic test identifies the presence of viral RNA from nasopharyngeal swabs of potentially infected people, with an analytic sensitivity of 1 × 10–2 TCID50/mL and an analytic specificity of 100% [9, 10]. This retrospective chart review study was approved by Stanford’s Institutional Review Board (IRB 55757).

### Patient population and data

We first identified all positive COVID-19 RT-PCR’s performed by Stanford laboratory, from March 4 to March 31, 2020. These represented a mix of patients seen at an SHC clinic/facility and those seen at external hospital systems that were using Stanford as reference laboratory. Date and location of testing, age at specimen collection, gender, race/ethnicity, insurance plan, and county of residence were electronically extracted from Stanford’s electronic medical record system. Only age and gender were available for patients tested at an external hospital; these patients were only included to assess how this broader group of patients compared to the more well-defined Covid patient population who sought testing at Stanford with respect to age and gender as a way to inform regional generalizability of the patients for whom we have more in-depth data.

**Fig. 1:**
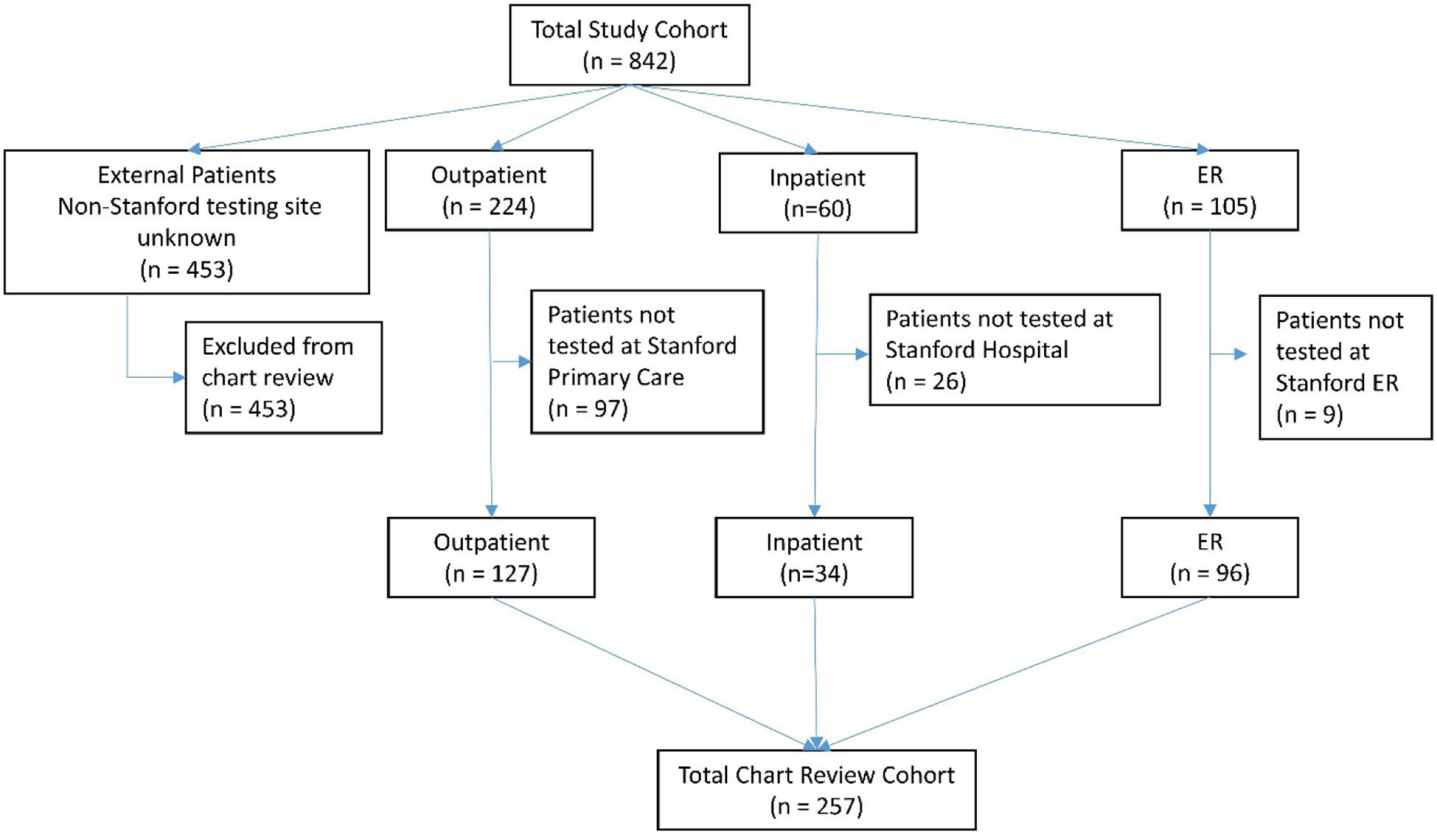
Flow chart depicting total study cohort of COVID-19 positive patients and how the total chart review cohort was achieved. ER, Emergency Room

We conducted a chart review on the subset whose tests were collected at SHC facilities, specifically, Stanford Hospital, Stanford ER, or one of ten Stanford primary care outpatient clinics including Stanford Express Clinic; patients whose test specimens were sent from a non-Stanford facility were excluded from the chart review (Fig. 1). Abstracted data included: potential source of exposure to COVID-19; symptoms; medical history; hospitalization; ICU admission; and death. Hospitalizations, ICU admission, and deaths were accessed through April 29, 2020. The chart review data were independently abstracted by two medical students using double data entry; a faculty physician then re-reviewed all charts and reconciled any inconsistencies.

### Data analysis

We calculated descriptive statistics overall and by location of presentation for all COVID-19 positive patients, including both those captured in external specimens sent from non-Stanford facilities as well as for the subset included in chart review, i.e. patients seen at a SHC clinic/facility. χ^2^ test, Fisher’s exact test, or Monte Carlo estimate for the Fisher’s exact test were used to compare differences between patients that presented at outpatient, ER, and inpatient.

We conducted logistic regression analyses on the chart review subset to assess factors associated with the odds of developing severe disease, defined as being hospitalized and/or death. Patients with insufficient data available to determine if they were hospitalized or died (i.e., no subsequent contact confirming recovery 10 days after positive test), were excluded from these analyses. In descriptive statistics and logistic regression models, we defined hypertension (HTN) to be in the absence of other cardiovascular disease (CVD) or stage 3+ chronic kidney disease (CKD), defined as pre-existing documentation of GFR < 60, in order to assess whether common HTN in the absence of more severe and often related complications/comorbidities, was associated with developing severe COVID-19.

Two regression models were fit separately examining associations with: 1) patient socio-demographics and disease severity; and 2) comorbidities and symptoms with disease severity. We combined statistically significant variables from (1) and (2) to form a semi-final model. Because there was strong clinical interest regarding the estimates for the remaining comorbidities and symptoms, we added each into the semi-final model individually to evaluate their associations; our goal with these analyses was to build upon the growing early literature to better describe patients most likely to develop severe disease. The final mode included all variables in the semi-final model plus any additional variables that were significant when added individually to it. Variables were not included in any of the models if > 10% of the data were missing (i.e, BMI and smoking) or they prevented model convergence (i.e., loss of taste and back pain). Confidence intervals excluding 1.0 are considered statistically significant in logistic regression models. Analyses were performed using SAS (version 9.4; SAS Institute, Inc., Cary, NC, USA).

## Results

### All COVID-19+ patients

Eight hundred forty-two local patients tested positive for COVID-19 in March 2020 through Stanford Health Care’s laboratory; over half of which (54%) were specimens sent from non-Stanford facilities (Fig. 1). There were significant differences in the demographics of positive COVID-19 patients by point of access to testing (Table 1) for all characteristics examined. The outpatient care setting had the highest proportion of women, Caucasians and commercially insured patients. Outpatients skewed toward younger patient with only 5% 75 years or older. In contrast, the inpatient setting had the highest proportion of men, age over 65 years, and Medicare-insured patients. The ER setting had the highest proportion of younger (43% less than 40 years), Hispanic (32%) and Medicaid-insured patients (23%). The external patients most closely resembled the age and sex distribution of the inpatients, suggesting these were hospitalized patients with more severe disease at time of diagnosis. Regardless of point of access, the vast majority (72%) of patients lived in one of the two counties closest to the main Stanford campus, Santa Clara or San Mateo; ER patients were most likely to be from these local counties (85% compared to 69 and 62% of outpatients and inpatients, respectively).

**Table 1:** Demographic Characteristics of All COVID-19 Patients Diagnosed in March 2020 at Stanford

Demographics	Total		Outpatient		Inpatient		ER		External	
	***N*** = 842	***%***^***b***^	***N*** = 224	***%***^***b***^	***N*** = 60	***%***^***b***^	***N*** = 105	***%***^***b***^	453	***%***^***b***^
Age (years)^***^										
< 40 years	228	*27*	78	*35*	4	*20*	45	*43*	93	*21*
40–54 years	207	*25*	58	*26*	8	*18*	25	*24*	113	*25*
55–64 years	169	*20*	53	*24*	9	*22*	13	*12*	90	*20*
65–74 years	115	*14*	24	*11*	8	*15*	7	*7*	75	*17*
75+ years	123	*15*	11	*5*	12	*25*	15	*14*	79	*18*
Sex^**^										
Female	391	*46*	128	*57*	23	*38*	51	*49*	189	*42*
Male	451	*54*	96	*43*	37	*62*	54	*51*	264	*58*
**Race/Ethnicity**^a***^										
Caucasian	156	*40*	98	*44*	16	*27*	42	*40*	–	–
Hispanic	78	*20*	31	*14*	13	*22*	34	*32*	–	–
Asian	68	*18*	40	*18*	12	*20*	16	*15*	–	–
Other	40	*10*	21	*9*	9	*15*	10	*10*	–	–
Missing	47	*12*	34	*15*	10	*17*	3	*3*	–	–
**Insurance**^a***^										
Commercial	211	*54*	132	*59*	20	*33*	59	*56*	–	–
Medicare	58	*15*	23	*10*	20	*33*	15	*14*	–	–
Medicaid	40	*10*	8	*4*	8	*13*	24	*23*	–	–
Uninsured	16	*4*	6	*3*	3	*5*	7	*7*	–	–
Missing	64	*17*	55	*25*	9	*15*	0	*0*		
**County of residence**^a***^										
Santa Clara	153	*39*	89	*40*	21	*35*	43	*41*	–	–
San Mateo	127	*33*	65	*29*	16	*27*	46	*44*	–	–
Alameda	54	*14*	36	*16*	10	*17*	8	*8*	–	–
San Francisco	17	*4*	11	*5*	2	*3*	4	*4*	–	–
Other	17	*4*	12	*5*	1	*2*	4	*4*	–	–
Missing	21	*5*	11	*5*	10	*17*	0	*0*	–	–

^a^Percentages calculated based on *n* = 389; the external referral group was omitted because the vast majority of their data was missing for these variables

^b^ Percentages may not add to 100 due to rounding

^*^ *P* ≤ 0.05

^**^ *P* ≤ 0.01

^***^ *P* ≤ 0.001

### Chart review subset

There were 257 patients in the chart review analysis: 127 (49%) outpatient; 34 (13%) inpatient; and 96 (37%) ER (Fig. 1). The demographic characteristics of the chart review subset (Table 2) were similar to that of the larger cohort (Table 1). Approximately 41% of patients had contact with someone they knew to have Covid-19, 25% had traveled recently and 6% lived in a group living situation (Table 2). The inpatient setting, however, had the highest proportion living in a communal setting (18%, compared to 3 and 6% tested in the outpatient and ER settings, respectively (*p* < 0.05)). The ER testing setting had the highest proportion of patients with known contacts (46%) and the outpatient testing setting had the highest proportion of recent travelers (31%), however, neither of these differed significantly by testing access point.

**Table 2:** Demographic Characteristics of COVID-19 Patients Diagnosed in March 2020 at Stanford: Chart review subset

Demographics	Total		Outpatient		Inpatient		ER	
	***N*** = 257	***%***^***a***^	***N*** = 127	***%***^***a***^	***N*** = 34	***%***^***a***^	***N*** = 96	***%***^***a***^
Age (years)^**^								
< 40 years	97	*38*	50	*39*	6	*18*	41	*43*
40–54 years	59	*23*	27	*21*	8	*24*	24	*25*
55–64 years	45	*18*	27	*21*	6	*18*	12	*13*
65–74 years	27	*11*	15	*12*	6	*18*	6	*6*
75+ years	29	*11*	8	*6*	8	*24*	13	*14*
**Sex**								
Female	129	*50*	69	*54*	13	*38*	47	*49*
Male	128	*50*	58	*46*	21	*62*	49	*51*
Race/Ethnicity^*^								
Caucasian	104	*41*	55	*43*	9	*27*	40	*42*
Hispanic	55	*21*	17	*13*	9	*27*	29	*30*
Asian	48	*19*	25	*20*	9	*27*	14	*15*
Other/Unknown	50	*20*	30	*24*	7	*21*	13	*14*
Insurance^***^								
Commercial	168	*65*	97	*76*	14	*41*	57	*59*
Medicare	43	*17*	17	*13*	13	*38*	13	*14*
Medicaid	30	*12*	7	*6*	4	*11*	19	*20*
Uninsured	16	*6*	6	*5*	3	*9*	7	*7*
**County of residence**								
Santa Clara/San Mateo	224	*87*	109	*86*	31	*91*	84	*88*
Other	33	*13*	18	*14*	3	*9*	12	*13*
**Potential transmission**								
Known contact^b^	105	*41*	48	*38*	13	*38*	44	*46*
Recent travel^c^	63	*25*	39	*31*	8	*24*	16	*17*
Communal Living situation^d*^	16	*6*	4	*3*	6	*18*	6	*6*

^a^Percentages may not add to 100 due to rounding error

^b^Close contact with known or suspected COVID-19 case

^c^Inside or outside the United States

^d^Includes shelter, dormitory, skilled nursing facility, assisted living facility, and homeless

^*^ *P* ≤ 0.05

^**^ *P* ≤ 0.01

^***^ *P* ≤ 0.001

The most common comorbidities in all three groups were BMI ≥ 30, lung conditions, HTN, and ever smoking. Frequency of comorbidities varied little between those tested in the outpatient and ER settings aside from diabetes (8% outpatient vs 18% ER). In contrast, inpatients had the highest proportion of every comorbidity examined, except for gastrointestinal (GI) conditions (Table 3). The largest difference was proportion with stage 3+ CKD as defined by history of GFR < 60, 21% of those tested in the inpatient setting but only 2% in the ER and outpatient settings. All differences were statistically significant (*p* < 0.05) except lung conditions, immunosuppressive conditions, neurologic conditions, and GI conditions (Table 3).

**Table 3:** Medical History of COVID-19 Patients Diagnosed in March 2020 at Stanford: Chart review subset

	Total		Outpatient		Inpatient		ER	
Known Medical History	***N*** = 257	***%***^***a***^	***N*** = 127	***%***^***a***^	***N*** = 34	***%***^***a***^	***N*** = 96	***%***^***a***^
Lung condition^b^	52	*20*	27	*21*	9	*27*	16	*17*
BMI 30 + ^***^	48	*19*	23	*18*	10	*29*	15	*16*
Hypertension alone^c***^	48	*19*	20	*16*	12	*35*	16	*17*
Ever smoker^***^	43	*17*	19	*15*	12	*35*	12	*13*
Diabetes^**^	35	*14*	10	*8*	8	*24*	17	*18*
Immunosuppressive condition^d^	19	*7*	10	*8*	5	*15*	4	*4*
Cardiovascular disease^e*^	19	*7*	9	*7*	6	*17*	4	*4*
Neurologic condition^f^	14	*5*	4	*3*	3	*9*	7	*7*
GI condition^g^	11	*4*	6	*5*	1	*3*	4	*4*
GFR <60^h***^	11	*4*	2	*2*	7	*21*	2	*2*
Liver condition^i*^	6	*2*	2	*2*	2	*6*	2	*2*

^a^ Percentages may not add to 100 due to rounding error

^b^ Lung conditions include asthma, COPD, chronic lung disease

^c^ Hypertension without history of cardiovascular disease or poor renal function

^d^ Immunosuppressive conditions include any of: chronic lung disease, cancer, organ transplant, immunosuppressive drugs, other indicator of immunosuppression

^e^ Cardiovascular disease includes CAD, myocardial infarction, or heart failure

^f^ Neurologic conditions include any of: Parkinson Disease, Alzheimer, cognitive impairment, other

^g^ GI condition includes IBD, IBS, and other

^h^ only includes those with GFR < 60 prior to diagnosis

^i^Liver condition includes Hepatitis B, Hepatitis C, Hepatic steatosis, cirrhosis, other

^*^ *P* ≤ 0.05

^**^ *P* ≤ 0.01

^***^ *P* ≤ 0.001

Table 4 shows the frequency of symptoms overall and by testing access point. The most common symptoms in all patients were cough (86%), fever/chills (76%), and fatigue (63%). There was more variation in frequency of other symptoms across patient groups. Specifically, outpatients had the highest proportion of any of the other symptoms except pleuritic chest pain, shortness of breath, GI symptoms, and back pain. ER patients were most likely to present with pleuritic chest pain (35% vs 24%; not significant), whereas inpatients were most likely to present with shortness of breath (65% vs 30–32%; *p* < 0.001) or GI symptoms (47% vs 22–24%; *p* < 0.05). Loss of taste and back pain/ache were the least common presenting symptoms investigated, 9 and 3%, respectively.

**Table 4:** Symptoms at Presentation for Testing of COVID-19 Patients Diagnosed in March 2020 at Stanford: Chart review subset

	Total		Outpatient		Inpatient		ER	
Symptoms	***N*** = 257	***%***	***N*** = 127	***%***	***N*** = 34	***%***	***N*** = 96	***%***
Cough	220	*86*	112	*88*	29	*85*	79	*82*
Fever/Chills^a***^	196	*76*	108	*85*	27	*79*	61	*64*
Fatigue, etc^b^*	162	*63*	91	*72*	21	*62*	50	*52*
Shortness of breath or Dypsnea on exertion^***^	90	*35*	38	*30*	22	*65*	30	*32*
Sore throat**	79	*31*	50	*39*	5	*15*	24	*25*
Runny nose or nasal congestion	74	*29*	43	*34*	5	*15*	26	*27*
Pleuritic chest pain, tightness^c^	72	*28*	30	*24*	8	*24*	34	*35*
Headache	69	*27*	42	*33*	9	*26*	18	*19*
GI symptoms^d*^	67	*26*	30	*24*	16	*47*	21	*22*
Loss of taste	23	*9*	17	*13*	1	*4*	5	*5*
Back pain, ache*	8	*3*	1	*1*	0	*0*	7	*7*

^a^Includes both known and subjective

^b^Includes fatigue, malaise, weakness, myalgias

^c^Includes all chest symptoms such as wheezing and chest congestion

^d^Includes nausea, vomiting, diarrhea

^*^ *P* ≤ 0.05

^**^ *P* ≤ 0.01

^***^ *P* ≤ 0.001

### Severe disease

A total of 41 patients experienced severe disease, defined as hospitalization or death. One outpatient died at home and four others were later hospitalized. Eight ER patients were later hospitalized, one of whom died. Two of the patients initially tested as inpatients died for a total of four deaths in the cohort. Table 5 shows the results of the logistic regression models. Five patients originally tested in the ER and two from the outpatient setting were lost to follow-up so were excluded from this analysis, the remaining 250 patients were included. The final model output indicates gender, race/ethnicity, and insurance were associated (or marginally associated) with developing severe disease after adjusting for comorbidities and symptoms. Specifically, male relative to female (OR = 2.2; 95% CI: 1.0, 4.7), Asian or Hispanic relative to Caucasian (OR = 4.8; 95% CI: 1.6, 14.2 and OR = 3.6; 95% CI: 1.1, 11.9, respectively), and Medicare insurance relative to commercial insurance (OR = 4.0; 95% CI: 0.9, 17.8) were the patient groups with statistically significant or marginally significant associations. Notably, age was not significantly associated after adjusting for insurance status, however, equally important is that 20% of patients aged 65+ had commercial insurance, thus older patients without commercial insurance had the highest risk of severe disease.

**Table 5:** Logistic Regression Models for Severe COVID-19 Disease (inpatient admission or death)

Patient Characteristics	Final Model		Additional Estimates	
	OR	95% CI	OR	95% CI
**Demographics**				
Age				
< 40 years (ref)	–	–		
40–54 years	1.4	0.4–4.4		
55–64 years	1.8	0.6–5.6		
65–74 years	3.0	0.6–14.4		
75+ years	0.8	0.1–5.4		
Sex				
Female (ref)	–	–		
Male	**2.2**	**1.0–4.7**		
Race/Ethnicity				
Caucasian (ref)	–	–		
Asian	**4.8**	**1.6–14.2**		
Hispanic	**3.6**	**1.1–11.9**		
Other or Unknown	2.3	0.7–7.1		
Insurance				
Commercial (ref)	–	–		
Medicare	**4.0**	**0.9–17.8**		
Medicaid	1.3	0.3–5.2		
Uninsured	2.3	0.5–9.8		
**Comorbidities**				
Cardiovascular disease^a^	**4.7**	**1.0–22.1**		
Hypertension alone^b^	**2.3**	**0.8–6.5**		
GFR < 60	**6.1**	**1.0–38.1**		
Neurologic condition^c^			2.3	0.5–10.8
Immunosuppressive condition^d^			1.3	0.2–7.4
Liver condition^e^			2.0	0.2–15.6
Diabetes			0.8	0.3–2.5
Lung condition^f^			0.6	0.2–1.6
GI condition^g^			0.3	0.0–4.0
**Symptoms**				
Shortness of breath or Dyspnea on exertion	**5.4**	**2.3–12.6**		
GI symptoms^h^	**3.3**	**1.4–7.7**		
Headache			1.0	0.4–2.3
Pleuritic chest pain, tightness^i^			0.9	0.4–2.2
Fatigue^j^			0.6	0.3–1.4
Sore throat			0.6	0.2–1.5
Runny nose or nasal congestion			0.4	0.2–1.2

^a^ Cardiovascular disease includes CAD, myocardial infarction, or heart failure

^b^ Hypertension without history of cardiovascular disease or poor renal function

^c^ Neurologic conditions include any of: Parkinson Disease, Alzheimer, cognitive impairment, other

^d^ Immunosuppressive conditions include any of: chronic lung disease, cancer, organ transplant, immunosuppressive drugs, other indicator of immunosuppression

^e^ Liver condition includes Hepatitis B, Hepatitis C, Hepatic steatosis, cirrhosis, other

^f^ Lung conditions include asthma, COPD, chronic lung disease

^g^ GI condition includes IBD, IBS, and other

^h^ Includes nausea, vomiting, diarrhea

^i^ Includes all chest symptoms such as wheezing and chest congestion

^j^ Includes fatigue, malaise, weakness, myalgias

CVD (OR = 4.7; 95% CI: 1.0, 22.1), HTN (OR = 2.3; 95% CI: 0.8, 6.5), and stage 3+ kidney disease (OR = 6.1; 95% CI: 1.0, 38.1), were all marginally statistically significantly related to development of severe disease in the final model (Table 5). Shortness of breath (OR = 5.5; 95% CI: 2.3, 13.0) and GI symptoms (OR = 3.3; 95% CI: 1.4, 7.7) were the only presenting symptom associated with developing severe disease. No other comorbidities or symptoms approached statistical significance.

## Discussion

This study examined the first cohort of ambulatory COVID-19 positive patients in the Silicon Valley region of California—one of the first communities in the United States to scale up community testing in response to early community spread. In exploring patient demographics, comorbidities and symptoms in relationship to their location of presentation and the development of severe disease, we reiterate the growing evidence of socio-demographic disparities in COVID-19’s impacts. Our work was motivated by desire to identify clinical predictors of progression to more severe disease, but instead we find that race/ethnicity and insurance predict risk of hospitalization at the same or similar order of magnitude as the most predictive comorbidities and symptoms. Thus, our data once again re-tells a story of racial inequity in health outcomes, but with specific local flavor. Silicon Valley’s diversity includes large representation of Asian and Latinx populations, and while these groups were slightly under-represented compared to the local population, they still each represented a fifth of our study population. Notably, we had too few African American patients in our cohort to meaningfully analyze.

Our findings are both consistent and distinct with similar work looking at the broader San Francisco Bay area, done by the Sutter Health Care system-- both studies demonstrate racial disparity in COVID-19, but highlight different affected minority communities [4]. Whereas theirs and prior work demonstrated 3-fold risk of hospitalization for African American COVID-19 patients, our cohort demonstrates a significant approximately 3-fold odds of hospitalization in Asian and Latinx patients. In particular, we believe this marked increased risk of hospitalization in Asian patients is a novel finding, made possible by the relatively large representation in our local population.

The fact that socio-demographic factors – including race/ethnicity and insurance type/status – were associated with severity likely reflects the confluence of multiple underlying disparities including social, economic, access, and behavioral factors [3, 4]. These might manifest as barriers to timely presentation to care and influence where patients eventually access care. This hypothesis is supported by our observation that the location of presentation (outpatient, inpatient, or emergency room), was most strikingly different by insurance status and race/ethnicity.

Latinx patients with COVID-19 were most likely to present to ER or inpatient settings. Patients with commercial insurance were most likely to present at an outpatient location while patients without insurance or with Medicaid were most likely to have their COVID-19+ status captured in the ER or once inpatient. Many factors might contribute to these differences, including familiarity with ways to rapidly access outpatient appointments, having a primary care physician, language and technical barriers to scheduling, cultural norms, perceptions of insurance requirements in different locations, and gaps in communication and knowledge of Stanford’s broadened access offered (i.e., accepting all-payers and uninsured) for outpatient COVID-19 testing.

There were notably fewer differences amongst location of presentation in terms of presenting symptom and co-morbidities; our study provides important descriptions of both, but also reiterates the diversity of symptoms that make clinical prediction a challenge for this disease. The two symptoms identified were shortness of breath/dyspnea and GI symptoms, which unsurprisingly (as respiratory distress is frequent trigger for hospital admission as is dehydration from severe vomiting and/or diarrhea) was more common in hospitalized patients (65 and 47%, respectively) than outpatient (30 and 24%, respectively) or emergency room (32 and 22%, respectively) presentations.

In our cohort, the comorbidities we observed to predict severe outcomes were consistent with the CDC’s recent update from July 13, 2020, and we re-confirm a strong association with underlying CVD (OR 4.7) and chronic renal disease (OR 6.1) [5]. In the same update, the CDC lists hypertension as having “mixed evidence” for severe disease. Our data, similarly found marginal evidence for hypertension alone as independent predictor of worse outcomes with odds ratio 2.3. We purposefully defined our hypertension variable to capture the more common cases of hypertension, uncomplicated by CVD or CKD. This is important, given hypertension is one of the most prevalent conditions in the US, affecting one-third of adults [11]. Our findings from our cohort of predominantly ambulatory COVID-19 patients supports parallel findings seen in the predominantly inpatient data from meta-analysis, and together reinforce that HTN alone may be an independent risk factor for developing severe disease [12]. Our lack of evidence of increased risk with asthma, neurologic conditions, or diabetes should more likely be interpreted to reflect our relatively small sample size, rather than evidence against their plausible association with severe COVID-19.

### Limitations and strengths

Despite broad testing criteria and early ramp-up of testing in our local system, the cohort included in our one-month chart-review was of relatively small sample size, which was particularly limiting in our ability to examine comorbidities with somewhat lower prevalence (e.g., neurologic conditions) and data with high missingness (e.g. BMI and tobacco use). Further, the limitations on testing availability in March led to a selection bias. In our clinical experience, when testing was limited, many younger and healthier patients were assumed positive and not tested. Outpatient testing was prioritized for older persons, which may bias some of our estimates.

Strengths of our study include our methodology of rigorous manual chart review, which allowed for comprehensive identification of both comorbidities and symptoms. The accuracy and completeness of these factors is beyond prior COVID-19 studies which has relied on diagnostic codes or used natural language processing to estimate COVID-19 symptomatology [2, 4]. Our race and ethnicity was data had relatively low rate of missingness, an issue that has been increasingly identified as a barrier to understanding the true extent of disparities in COVID-19 [13, 14]. The study also had high follow-up for outcomes of disease severity at 97% of all patients.

## Conclusions

When and how care is accessed and the outcomes for COVID-19 severe disease is affected by ethnicity and insurance type. We reiterate the disproportionate impact of COVID-19 on minority populations and specifically find that in a largely ambulatory population, in a region with large Asian and Latinx representation, that both of these race/ethnicity groups were associated with more severe cases of COVID-19. We also find further marginal evidence to support the to-date uncertain association of hypertension (independent of renal or more severe cardiovascular disease) with more severe COVID-19 disease [5].

## Data Availability

The datasets generated and/or analyzed during the current study are not publicly available because they are governed by the data stewards of Stanford Health Care. They may be available upon reasonable request pending review by the Privacy Office.

## Abbreviations

BMI: Body mass index
CDC: Centers for Disease Control
CKD: Chronic kidney disease
CVD: Cardiovascular disease
ER: Emergency room
GI: Gastrointestinal
GFR: Glomerular filtration rate
HTN: Hypertension
OR: Odds ratio
RT-PCR: Real-time reverse transcription polymerase chain reaction
SHC: Stanford Health Care

## References

[1] Centers for Disease Control and Prevention. Updated Guidance on Evaluating and Testing Persons for Coronavirus Disease 2019 (COVID-19). Available at: https://emergency.cdc.gov/han/2020/han00429.asp. Accessed 13 July 2020.

[2] Medium. Estimating the feasibility of symptom based classification of COVID-19. Available at: https://medium.com/@nigam/estimating-the-feasibility-of-symptom-based-classification-of-covid-19-cdba1e1f1950. Accessed 20 Mar 2020.

[3] Hooper MW, Nápoles AM, Pérez-Stable EJ (2020). COVID-19 and racial/ethnic disparities. J Am Med Assoc.

[4] Azar KMJ, Shen Z, Romanelli RJ (2020). Disparities in outcomes among COVID-19 patients in a large health care system in California. Health Aff.

[5] Centers for Disease Control and Prevention. Evidence used to update the list of underlying medical conditions that increase a person’s risk of severe illness from COVID-19. Available at: https://www.cdc.gov/coronavirus/2019-ncov/need-extra-precautions/evidence-table.html. Accessed 13 July 2020. 34009770

[6] Artandi M, Thomas S, Shah NR, Srinivasan M. Rapid system transformation to more than 75% primary care video visits within three weeks at Stanford: response to public safety crisis during a pandemic. NEJM Catal Innov Care Deliv. 2020. 10.1056/CAT.20.0100.

[7] United States Census Bureau. U.S. Census Bureau QuickFacts: Santa Clara County, California. Available at: https://www.census.gov/quickfacts/santaclaracountycalifornia. Accessed 24 July 2020.

[8] United States Census Bureau. U.S. Census Bureau QuickFacts: San Mateo County, California. Available at: https://www.census.gov/quickfacts/fact/table/sanmateocountycalifornia/PST045219. Accessed 24 July 2020.

[9] Centers for Disease Control and Prevention. Health, United States, 2018 – Data Finder. Available at: https://www.cdc.gov/nchs/hus/contents2018.htm. Accessed 30 Mar 2020.

[10] Hologic. SARS-CoV-2 Assay (Panther Fusion® System). Available at: https://www.hologic.com/sites/default/files/2020-04/AW-21159-001_003_01_0.pdf. Accessed 25 July 2020.

[11] Centers for Disease Control and Prevention. Selected health conditions and risk factors, by age: United States, selected years 1988–1994 through 2015–2016. Available at: https://www.cdc.gov/nchs/data/hus/2018/021.pdf. Accessed 29 Oct 2019.

[12] Pranata R, Lim MA, Huang I, Raharjo SB, Lukito AA (2020). Hypertension is associated with increased mortality and severity of disease in COVID-19 pneumonia: a systematic review, meta-analysis and meta-regression. J Renin-Angiotensin-Aldosterone Syst.

[13] Oppel RA, Gebeloff R, Lai KKR, Wright W, Smith M (2020). The Fullest Look Yet at the Racial Inequity of Coronavirus. The New York Times.

[14] Hellmann J (2020). Frustrations grow over incomplete racial data on COVID-19 cases, deaths.

